# Endonasal Endoscopic Approach for Bone Spicule Removal: A Case Report

**DOI:** 10.1155/crot/8857565

**Published:** 2026-02-03

**Authors:** Utku Kubilay, Kaan Işıklar

**Affiliations:** ^1^ Department of Otolaryngology–Head and Neck Surgery, Health Sciences University İzmir Tepecik Training and Research Hospital, İzmir, Turkey; ^2^ Department of Otolaryngology–Head and Neck Surgery, Izmir Sehir Hastanesi, Izmir, Turkey

**Keywords:** endoscopy, minimally invasive surgical procedures, osteotomy, postoperative complications, rhinoplasty

## Abstract

During rhinoplasty surgery, tiny bone spicules (BSs) can form, particularly if blunt tools are used. If these BSs are not noticed and removed intraoperatively, they can cause cosmetic problems and nasal swelling postoperatively. A patient who underwent open rhinoplasty 2 years ago presented with a painless swelling causing cosmetic problems near the left medial canthus. A BS was detected by paranasal sinus computerized tomography and removed using an endonasal endoscopic approach (EEA). At 13 months of postoperative follow‐up, the patient reported no complaints. The use of sharpened osteotomes or powered instruments, capable of creating the precise and desired osteotomies, is crucial to prevent the formation of BS. Detecting these BS intraoperatively is also desirable. Administering intravenous corticosteroids, such as prednisolone (1 mg/kg), maintaining hypotensive anesthesia, applying ice packs, and elevating the head 30° during surgery can help reduce edema and aid in the identification of BS. Saline irrigation and surgical field aspiration can facilitate the removal of BS. Final inspection and palpation of the nose from different angles under proper lighting are essential. In cases where BSs are overlooked, an EEA with minimal dissection and no skin scar may be preferred for their extraction. EEA is a minimally invasive, practical, and successful treatment option for BS extraction.

**Main Points**
• BSs form during rhinoplasty, causing cosmetic and functional problems if not detected.
• A BS‐induced nasal swelling was successfully removed using a minimally invasive EEA.
• Use sharp tools, corticosteroids, and proper surgical techniques to prevent BS formation.
• EEA is effective for the removal of BS with minimal dissection and without skin scarring.

## 1. Introduction

Unwanted localized masses can develop in the nose as early or late complications of rhinoplasty. These may be caused by bone spicules (BSs) left behind during osteotomies, which can undergo neo‐osteogenesis over time and gradually enlarge as the surrounding tissue heals [1].

These masses generally present along osteotomy lines but can occasionally migrate to different areas of the nose. While they tend to cause esthetic problems predominantly, they can occasionally cause functional problems depending on their location and size.

In this paper, we present a case of endonasal endoscopic surgical excision of a mass associated with BS that formed on a previous osteotomy line after rhinoplasty.

## 2. Case Presentation

A 35‐year‐old male patient, who underwent open rhinoplasty 2 years ago, presented to our clinic with progressive swelling on the left side of the nose for the past year. The patient did not report a history of wearing glasses or trauma to the nose after surgery. In addition, the patient had no difficulty breathing through the nose and had discontinued follow‐up after the 3‐month examination.

**FIGURE 1:**
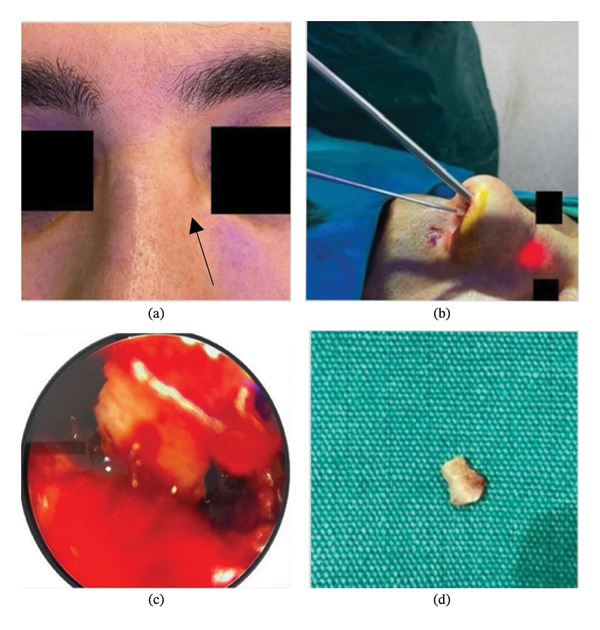
(a) The black arrow demonstrates the appearance of a nasal mass on physical examination. (b) Endonasal endoscopic approach. (c) Endoscopic view of the bone spicule before extraction. (d) Removed bone spicule.

External physical examination revealed a slight subluxation of the nasal caudal septum from the nasal spine toward the right side, a slight deviation of the nasal axis to the right, starting from the rhinion, and a 3 mm firm, fixed ovoid subcutaneous mass, approximately 7 mm from the left medial canthus. The mass had a smooth surface and was not tender (Figure 1).

**FIGURE 2:**
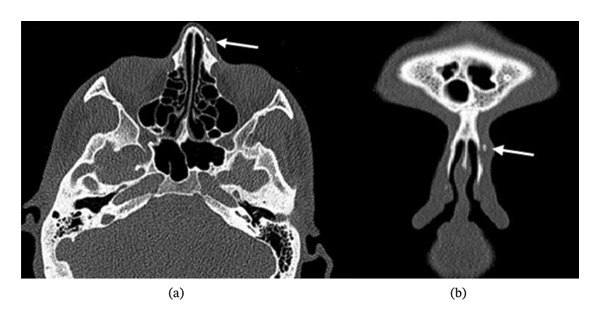
(a) Paranasal CT, axial imaging plane. (b) Paranasal CT, coronal imaging plane (white arrow indicates the bone spicule).

The endoscopic examination showed a slight deviation to the left, with no nasal passage obstruction. There was no relevant medical or medication use history, and laboratory tests were unremarkable. The tomography (CT) of the paranasal sinuses was conducted using coronal and axial imaging planes (Figure 2).

A 3‐mm free bone fragment was seen at the junction of the previous lateral and medial osteotomy lines on the left side. Removal of the mass was planned using a minimally invasive endonasal endoscopic approach (EEA). Infiltration anesthesia was administered externally around the mass and internally at the caudal end of the left nasal bone using 1 mL of 20 mg of lidocaine hydrochloride and 0.0125 mg of epinephrine.

An ENT endoscope of 0°, 4 × 175 mm (Richard Wolf GmbH, Knittlingen, Germany), was used for the procedure. A mucosal incision, approximately 1 cm long, was made along the caudal border of the left nasal bone. Using an elevator, a tunnel was created, approximately 1 cm wide, in a vertical plane. The bone fragment was located approximately 23 mm from the caudal end of the nasal bone. Hemostasis was achieved using gauze pads soaked in 1 mg/10 mL (1:10,000) epinephrine. The bone fragment was extracted under endoscopic visualization using otologic alligator forceps (Olympus/Gyrus Hartmann Alligator, Center Valley, PA, USA). Intraoperative endoscopic visualization and removal of the BS are demonstrated (Figure 1). The incision site was left unsutured, and the operation was completed without complications. The patient did not develop complications during the 13‐month follow‐up.

## 3. Discussion

Following rhinoplasty, localized masses can form as early complications due to simple soft tissue edema and hematomas. Late complications can include lipogranulomas, epidermoid inclusion cysts, tumefactive cartilage proliferation, mucous cysts, and BS [2]. The differential diagnosis of these lesions is based on the patient’s history, physical examination, and imaging, with paranasal sinus CT scans being the preferred method. Magnetic resonance imaging can also be requested for further imaging. These radiological examinations provide critical information about the size, anatomical location, relationship with surrounding tissues, encapsulation, vascular connections, and whether it is solid or cystic [3]. In addition, they can reveal intranasal and intracranial extensions, facilitating surgical planning and increasing safety.

BSs are common repeated osteotomies that deviate from the desired line, with bone characteristics playing a crucial role in fragment formation [4].

While 15‐degree medial osteotomies create more accurate and even fractures, 0‐degree medial osteotomies may cut thicker bone, resulting in the formation of thicker BSs that attach to the mobile segment [5].

Sharpening tools before performing an osteotomy reduces the risk of unwanted BS formation. Using powered instruments, such as Piezotome, motorized saws, and drills, minimizes this risk by creating precise incisions at desired locations [6].

During open rhinoplasty, hump resections are performed under direct vision. Elevation of the skin in this region facilitates irrigation and aspiration. After lateral and medial osteotomies, which are performed percutaneously or internally, there is a higher risk of overlooking BS. Due to the higher bone density in the triangular area near the medial side of the nasion region, it is important to avoid performing osteotomies in this area, as the risk of bleeding and BS formation may increase during the procedure [4]. These fragments typically remain close to osteotomy lines but can occasionally migrate to different areas of the nose. Osteotomies are usually performed at the end of surgery, when edema and hematomas make bone fragments difficult to detect.

Intraoperative measures to reduce edema and bleeding, such as intravenous corticosteroids (1 mg/kg/day, not exceeding 80–100 mg of sodium methylprednisolone), ice packs, controlled hypotension, and elevation of the head at 30°, may aid in the detection of BS. Careful palpation of the osteotomy lines, inspection from various angles under adequate illumination, and irrigation with saline, followed by aspiration, can facilitate the identification and removal of BS [7].

BSs can be removed through an external skin incision, open rhinoplasty, closed rhinoplasty, or endoscopically. External approach, although straightforward, carries a risk of scarring, which is often unacceptable for patients seeking cosmetic surgery. Open rhinoplasty involves a columellar incision and extensive dissection, which may be undesirable for small masses. The closed technique requires widening the dissection area for sufficient visibility, making it challenging to locate and extract the BS.

Endoscopic excision is recommended due to its minimal invasiveness and the lack of a skin incision. Even small BSs can be identified and removed using a 0° endoscope and otologic alligator forceps. This technique is facilitated externally by visually observing and manually palpating the BS, requiring minimal dissection for extraction.

Endoscopic approaches allow for rapid recovery. However, the disadvantages of the endoscope include the susceptibility to frequent contamination and impaired visibility due to bleeding in a limited space. Regular irrigation and the use of surgical pledgets soaked in 20 mg of lidocaine hydrochloride and 0.0125 mg of epinephrine can mitigate this problem.

Intraoperative measures to prevent BS include the administration of intravenous methylprednisolone (1 mg/kg), performing surgery with the head elevated at 30°, ensuring controlled hypotension anesthesia, applying ice during surgery, using sharp instruments during osteotomies, and irrigating and aspirating the surgical field. Before completing surgery, it is important to inspect and palpate the nose from different angles under proper illumination. If BS is overlooked as a complication of rhinoplasty, it can be removed safely and aesthetically using the EEA, which is a highly practical and successful treatment option for BS detected after rhinoplasty.

## 4. Conclusion

BSs may be encountered as rare late findings after rhinoplasty and can be associated with cosmetic or functional concerns if unrecognized. Attention to surgical technique and careful intraoperative assessment may help reduce the likelihood of their development. When BSs are identified during postoperative follow‐up, endonasal endoscopic removal may represent a minimally invasive management option in selected cases.

**Nomenclature**
BS: Bone spicule
EEA: Endonasal endoscopic approach

## Funding

No funding was received for this study.

## Ethics Statement

Our institution does not require ethical approval for reporting individual cases or case series. This case report study complies with the Declaration of Helsinki.

## Consent

Written informed consent was obtained from the participant prior to inclusion in the study.

## Conflicts of Interest

The authors declare no conflicts of interest.

## Data Availability

The data that support the findings of this study are available on request from the corresponding author. The data are not publicly available due to privacy or ethical restrictions.
